# Avapritinib monotherapy induces rapid and deep remission of heavily treated, *KIT* D816H-mutated t(8;21) acute myeloid leukemia, a case report and literature review

**DOI:** 10.1007/s00277-025-06388-w

**Published:** 2025-06-27

**Authors:** Ted M. Getz, Kamila Bakirhan, Stuart Seropian, Rory M. Shallis

**Affiliations:** 1 Department of Internal Medicine, Section of Hematology, Yale University, New Haven, CT USA; 2 Department of Hematology and Oncology, Danbury Hospital, Nuvance Health, Danbury, CT USA

**Keywords:** Acute myeloid leukemia, KIT mutations, t(8;21), Avapritinib, Case report

## Abstract

Acute myeloid leukemia (AML) with t(8;21) is a subset of core binding factor AML and is considered to be favorable risk disease in patients receiving intensive cytarabine based chemotherapy. However, relapse remains a significant clinical challenge. Mutations in *KIT*, which frequently co-occur in t(8;21) AML, have been associated with worse relapse free and overall survival. Avapritinib is a novel tyrosine kinase inhibitor targeting KIT mutations that is approved for systemic mastocytosis but doesn’t currently have an established role in the treatment of AML. We present a case of a patient with extensively treated KIT D816H-mutated t(8;21) AML who experienced relapse after an allogeneic hematopoietic stem cell transplant and achieved a deep remission rapidly with avapritinib monotherapy. This case highlights the potential role of avapritinib as a targeted therapy for relapsed t(8;21) AML with KIT mutations, warranting further clinical investigation.

## Introduction

Approximately 7% of acute myeloid leukemia (AML) harbors t(8;21), a core binding factor (CBF) lesion that leads to the RUNX1/RUNX1T1 (AML1/ETO) fusion gene and consequently the translation of an abnormal transcription factor that induces leukemogenesis [1]. Although AML with t(8;21) is over-represented in younger patients with AML and is associated with excellent response to cytarabine based chemotherapy, 40–50% of patients will relapse when treated with chemotherapy alone [2]. Furthermore, AML with t(8;21) frequently harbors mutations in *KIT* that lead to constitutive KIT activation, mediate activation of downstream signaling pathways involved in cell proliferation, differentiation, and survival, including the PI3K, JAK/STAT, MAPK, and Src kinase pathways, ultimately associating in some studies with inferior relapse free survival and overall survival (OS) [3–5].

KIT inhibitors have been studied in AML, namely the non-selective KIT inhibitor dasatinib in combination with standard intensive induction therapy for *KIT-*mutated CBF-AML and has yielded encouraging results [6, 7]. However, robust data for the use of gemtuzumab ozogamicin for CBF-AML and the lack of randomized data in support of a true benefit for KIT inhibition has limited broad uptake of this approach. More selective and potent KIT inhibitors are now available, including avapritinib, which is currently approved for the treatment of advanced systemic mastocytosis based on the results of the PATHFINDER and EXPLORER trials [8, 9]. These data provide the rationale for the use of avapritinib for use in the management of *KIT*-mutated AML. Here we describe the case of a 31-year-old female with AML harboring t(8;21) and a *KIT* D816H mutation that relapsed following allogeneic hematopoietic stem cell transplant (alloHSCT) and was successfully treated with avapritinib monotherapy.

## Patient presentation

A 31-year-old woman presented with cough, fever, weakness, and menorrhagia. She was tachypneic with a respiratory rate in the 20 breaths/min and tachycardic with a heart rate in the 114 beats/minute, but was hemodynamically stable without the need for supplementary oxygen. Physical examination revealed a pale, obese woman, with lungs that were clear to auscultation and without ecchymosis or petechiae. She was found to have a white blood cell (WBC) count of 23k/uL with 67% circulating blasts, anemia (hemoglobin 2.7 g/dL), and thrombocytopenia (platelet 37k/uL). LDH was elevated to 502U/L (normal range 122-241U/L), and there was no evidence of tumor lysis or disseminated intravascular coagulation with creatinine, phosphate, calcium, uric acid, PT and PTT all within normal limits. CT scan of the chest revealed patchy ground glass airway opacities in the upper and lower lobes bilaterally concerning for multifocal pneumonia, and she was started on vancomycin and zosyn. A bone marrow evaluation revealed a hypercellular marrow (90%) with 69% blasts that were CD33+. Fluorescence in situ hybridization (FISH) revealed t(8;21) and 4 copies of *KMT2A* in 12% of cells. Polymerase chain reaction (PCR) for AML-ETO was not sent at this time. Next generation sequencing (NGS) revealed a *KIT* D816H mutation with a variant allele frequency (VAF) of 44%.

**Fig. 1:**
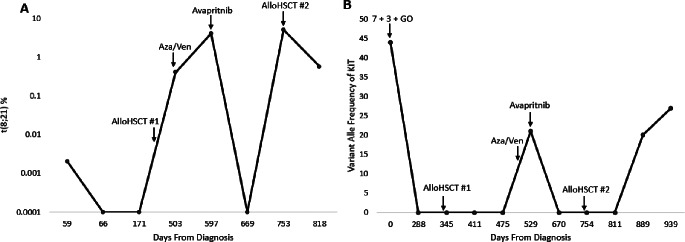
**(A)** Percent of cells with t(8;21) by PCR over time and **(B)** Variant alle frequency of KIT over time

The patient was induced with 7 + 3 with gemtuzumab ozogamicin and achieved a complete remission (CR) with FISH negative for t(8;21), although PCR still positive for AML-ETO transcripts (Fig. 1). After 2 cycles of high dose cytarabine (HiDAC) consolidation peripheral blood was negative for AML-ETO by PCR and *KIT* by NGS. However, AML-ETO was re-detected by PCR after second cycle of HiDAC and she ultimately underwent a matched related donor alloHSCT from her sister with myeloablative conditioning with busulfan and fludarabine. Bone marrow evaluations on day + 30, +90, and + 180 showed continued remission with t(8;21) negative by FISH as well as AML-ETO negative by PCR and *KIT* negative by PCR and NGS.

However, on day + 193 post-alloHSCT, 2% blasts were detectable in peripheral blood with t(8;21) and *KIT* D816H (VAF of 21%) also re-detected by NGS. A bone marrow evaluation was deferred as considered peripheral blood blasts rapidly increased to 60%. Relapsed/refractory therapy was initially deferred due to recurrent infections including Pantoea agglomerans bacteremia, vulvar HSV, and mucopurulent bacterial sinusitis that were treated with ceftriaxone and foscarnet, but on day + 263 she eventually started azacitidine and venetoclax. A post-cycle 1 marrow revealed a hypercellular marrow (40–50% cellular) with 10% blasts on an aspirate cell count. Persistent AML was likewise revealed after a cycle 2 marrow aspirate with 7–10% blasts and was for 3rd line therapy.

Avapritinib 100 mg daily was started a and bone marrow biopsy 30 days later revealed CR without measurable residual disease by flow, PCR for AML-ETO and NGS for *KIT.* This favorable response allowed a second alloHSCT 112 days after achieving CR from a 9/10 HLA mismatched unrelated donor after fludarabine and melphalan with post-transplant cyclophosphamide. Avapritnib was held at the time of transpant. Her 2nd post-alloHSCT course was complicated by stage 2 grade 1 cutaneous graft-versus-host disease and suspect grade 1 liver graft-versus-host disease due to elevations in transaminases that resolved with low-dose tacrolimus. Repeat marrow evaluation on day + 31 after 2nd alloHSCT confirmed CR with FISH and molecular MRD-negativity as well as 100% donor chimerism.

Unfortunately, bone marrow biopsy on day + 108 showed recurrent AML with 7% blasts on aspirate count. FISH showed that 50% cells harbored t(8;21) and PCR was AML-ETO was not done. NGS revealed recurrence of her *KIT* D816H mutation with a VAF of 20%. Avapritinib 100 mg daily was re-initiated on day + 122; however, the patient did not respond. A repeat bone marrow biopsy done on day + 157 showed an increase in blasts to 28% with an increase in *KIT* D816H VAF to 27%. The decision was made to pursue intensive chemotherapy and avapritnib was continued. Prior to admission for chemotherapy the patient developed a subdural hematoma complicated by brain stem herniation in the setting of thrombocytopenia with plt count < 20k/uL and died on day + 211 after 2nd alloHSCT.

## Discussion/Literature review

*KIT* mutations are present in 20–45% of patients with CBF-AML, including nearly half of patients with AML with t(8;21) and represents a promising target in AML [10]. Based on the activity of avapritinib for *KIT* D816V-mutated advanced systemic mastocytosis and case reports of efficacy in hematologic malignancies, the patient was treated with avapritinib [11, 12]. This led to a molecular-MRD-negative CR and the ability to proceed to a second alloHSCT.

On review of the literature, we were able to find four additional case reports or case series of patients with AML with t(8;21) and *KIT* mutation who were treated with avapritinib, although the largest (*n* = 20) was restricted to use as a post-alloHSCT MRD-directed therapy, a minority (*n* = 3) of patients for whom avapritinib was used in overt relapse, and none of which received avapritinib monotherapy (Table 1) [12–14]. Our patient is the only one in the available literature that has achieved an MRD-negative CR with avapritinib monotherapy in the relapsed/refractory setting, including after venetoclax exposure/failure, a situation notorious for lack of efficacy with subsequent therapy.

**Table 1:** Case reports of avapritinib used in patients with AML

Population	KIT Mutation	Sex	Age Range	Number of Patients	Therapy	Response	Reference
MRD positive AML with t(8;21) and KIT mutation after AlloHSCT	50% D816, 20% N822, 10% D816 + N822, and 20% other	15 men and 5 women	4 to 41 Years (2 ≤ 18 years and 18 ≥ 18 years)	20	Avapritinib 100 mg daily if weight ≥ 50 kg and 50 mg daily if weight < 50 kg	20% became MRD negative; 25% with ≥ 2 log reduction in t(3;21) transcript; 60% with ≥ 1 log reduction in t(3;21) transcript.	Kong et al. 2023 [1]
AML with t(8;21) and KIT mutation relapsed after AlloHSCT	D816I	Women	8 years old	1	Avapritinib 200 mg daily	t(8;21) negativity achieved 42 days after starting treatment.	Xue et al. 2022 [2]
Relapsed/Refractory AML with t(8;21) and KIT mutation previously treated with intensive chemotherapy	50% D816V and 50% D816Y	3 men and 1 women	19 to 52 years old	4	Avapritinib 100 mg daily + Venetoclax; Avapritinib 15 mg daily + Azacitidine + Venetoclax; Avapritinib 200 mg in 2 patients	2 MRD negative complete remission and 2 MRD positive complete remissions	Yin et al. 2022 [3]
AML with t(8;21) and KIT mutation after AlloHSCT MRD positive or MRD negative	33% N822I, 17% D816Y, 50% exon 17	5 men and 1 women	4 to 12 years old	6; 4 MRD positive and 2 MRD negative	Avapritinib 25-100 mg daily	3 of 4 patients who were MRD positive converted to MRD negative. 2 RMD negative patients remained MRD negative	Wang et al. 2023 [4]

Avapritinib appears to be a promising agent for the treatment of *KIT*-mutated CBF-AML with a well-studied and favorable safety profile. The response in this case clearly illustrates a need for further study. There are two ongoing phase II clinical trials in China evaluating avapritinib in relapsed/refractory *KIT*-mutated AML (NCT05821738 and NCT05821738), although there are no trials open with in the United States (US). We hope this informative case, believed to be the first of reported use of avapritinib for AML in the US, empowers providers to consider its use for similar patients who have no true standard of care in the relapsed/refractory setting that often requires the administration of therapies with less attractive risk: benefit profiles.

## Data Availability

No datasets were generated or analysed during the current study.
